# Author Correction: Tuning Li occupancy and local structures for advanced Co-free Ni-rich positive electrodes

**DOI:** 10.1038/s41467-025-58942-9

**Published:** 2025-04-15

**Authors:** Hang Li, Hao Liu, Shunrui Luo, Jordi Arbiol, Emmanuelle Suard, Thomas Bergfeldt, Alexander Missyul, Volodymyr Baran, Stefan Mangold, Yongchao Zhang, Weibo Hua, Michael Knapp, Helmut Ehrenberg, Feng Pan, Sylvio Indris

**Affiliations:** 1 School of Advanced Materials, Peking University Shenzhen Graduate School, Shenzhen, China; 2 Institute for Applied Materials (IAM), Karlsruhe Institute of Technology (KIT), Eggenstein-Leopoldshafen, Germany; 3 Catalan Institute of Nanoscience and Nanotechnology (ICN2), CSIC and BIST, Campus UAB, Bellaterra, Barcelona, Catalonia Spain; 4 ICREA, Barcelona, Catalonia Spain; 5 Institute Laue-Langevin (ILL), Grenoble, France; 6 CELLS-ALBA Synchrotron, Barcelona, Spain; 7 Deutsches Elektronen-Synchrotron (DESY), Hamburg, Germany; 8 Institute for Photon Science and Synchrotron Radiation, Karlsruhe Institute of Technology (KIT), Eggenstein-Leopoldshafen, Germany; 9 School of Chemical Engineering and Technology, Xi’an Jiaotong University, Xi’an, Shanxi China; 10 Applied Chemistry and Engineering Research Centre of Excellence (ACER CoE), Université Mohammed VI Polytechnique (UM6P), Ben Guerir, Morocco

**Keywords:** Batteries, Batteries, Batteries

Correction to: *Nature Communications* 10.1038/s41467-025-57063-7, published online 5 March 2025

In this article the e-mail address of the corresponding author Sylvio Indris was incorrect. The original article has been corrected.

