# Supplementary material for: Tuning Li occupancy and local structures for advanced Co-free Ni-rich positive electrodes
Source: Nat Commun. 2025 Mar 5;16:2203. doi: 10.1038/s41467-025-57063-7 (PMC11882807; doi:10.1038/s41467-025-57063-7)
Supplement: Supplementary file 1 — Supplementary Information [file 41467_2025_57063_MOESM1_ESM.pdf]

Tuning Li Occupancy and Local Structures for Advanced Co-Free Ni-Rich Positive Electrodes

Hang Li[1,2*], Hao Liu[2], Shunrui Luo[3], Jordi Arbiol[3,4], Emmanuelle Suard[5], Thomas Bergfeldt[2], Alexander Missyul[6], Volodymyr Baran[7], Stefan Mangold[8], Yongchao Zhang[3], Weibo Hua[9], Michael Knapp[2], Helmut Ehrenberg[2], Feng Pan[1*], Sylvio Indris[2,10*]

[1]School of Advanced Materials, Peking University, Shenzhen Graduate School, Shenzhen 518055, China.

[2]Institute for Applied Materials (IAM), Karlsruhe Institute of Technology (KIT), 76344 Eggenstein-Leopoldshafen, Germany.

[3]Catalan Institute of Nanoscience and Nanotechnology (ICN2), CSIC and BIST, Campus UAB, Bellaterra, 08193 Barcelona, Catalonia, Spain.

[4]ICREA, Pg. Lluís Companys 23, 08010 Barcelona, Catalonia, Spain.

[5]Institute Laue-Langevin (ILL), BP 156, 71 Avenue des Martyrs, 38042 Grenoble, France.

[6]CELLS-ALBA Synchrotron, 08290 Cerdanyola del Vallès, Barcelona, Spain.

[7]Deutsches Elektronen-Synchrotron (DESY), 22607 Hamburg, Germany.

[8]Institute for Photon Science and Synchrotron Radiation, Karlsruhe Institute of Technology (KIT), 76344 Eggenstein-Leopoldshafen, Germany

[9]School of Chemical Engineering and Technology, Xi'an Jiaotong University, Xi'an, Shanxi 710049 China.

[10]Applied Chemistry and Engineering Research Centre of Excellence (ACER CoE), Université Mohammed VI Polytechnique (UM6P), Lot 660, Hay Moulay Rachid, Ben Guerir, 43150, Morocco.

Corresponding authors: HL: 2206393335@pku.edu.cn; FP: panfeng@pkusz.edu.cn; SI: sylvio.indirs@kit.edu.

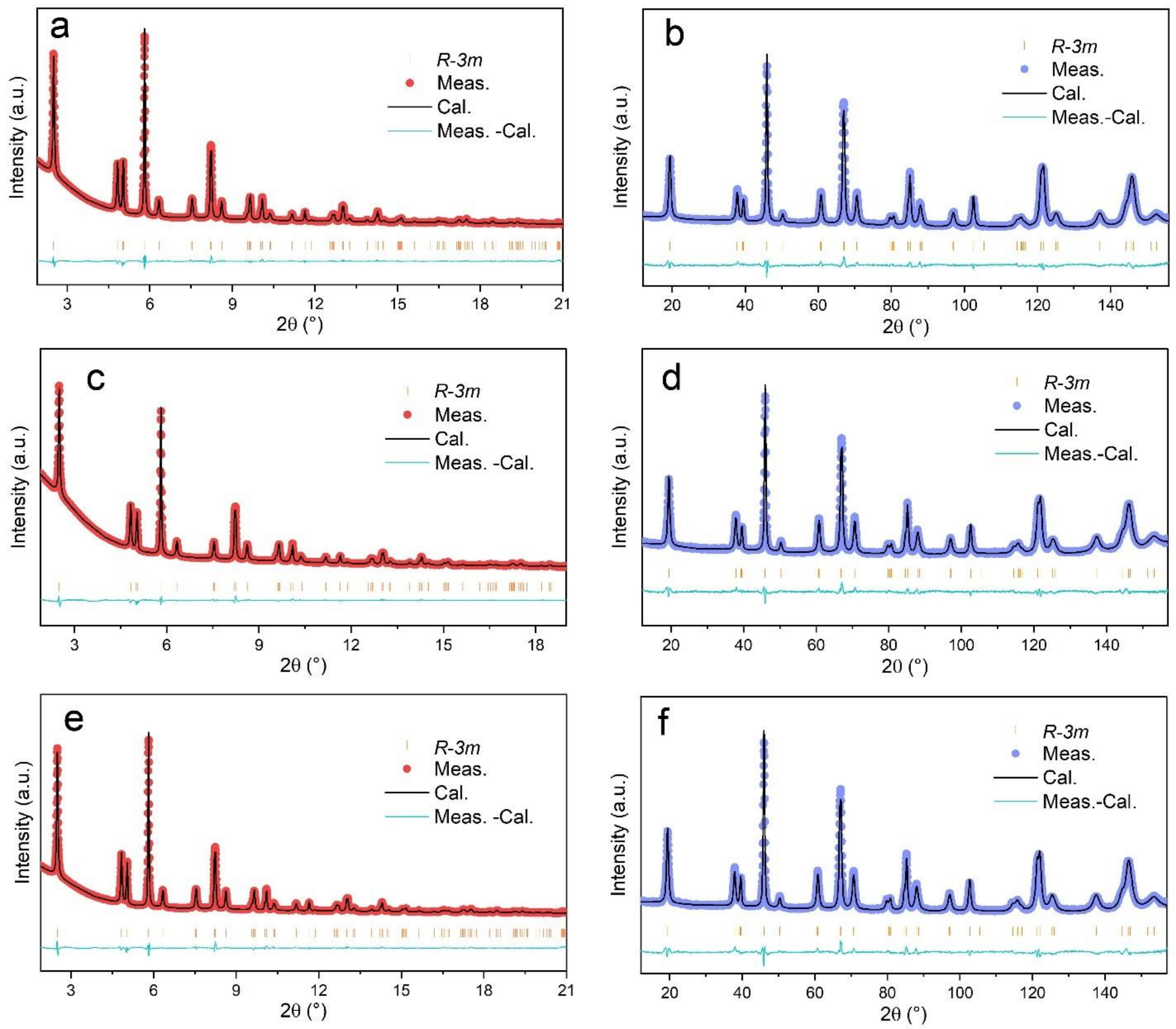

Fig. S1 Synchrotron XRD (λ = 0.2073 Å) and ND (λ = 1.594 Å) patterns and combined Rietveld refinement results of HD-LNMO-102 (**a**, **b**), 108 (**c**, **d**), and 114 (**e**, **f**).

Supplementary Note 1

During the combined refinement, the unit cell parameters, size broadening, scale factor, zero shift, isotropic atomic displacement parameters, the oxygen *z* coordinate, and site occupancy factors were refined in parallel. In the structural model, additional Ni was allowed on the Li site in Li layers and also for additional Li on the Ni site in TM layers, so the overall Ni includes Ni in Li layers and Ni in TM layers; The overall Li also includes two parts: Li in Li layers and Li in TM layers. The overall values for Ni and Li are calculated based on three constrains: the sum of Li and TM is 2, corresponding to the full occupancy of the TM and Li sites; the ratio of Li/TM, which is determined by the ICP-OES measurement; the ratio of Ni/TM, which is also given from the ICP-OES results.

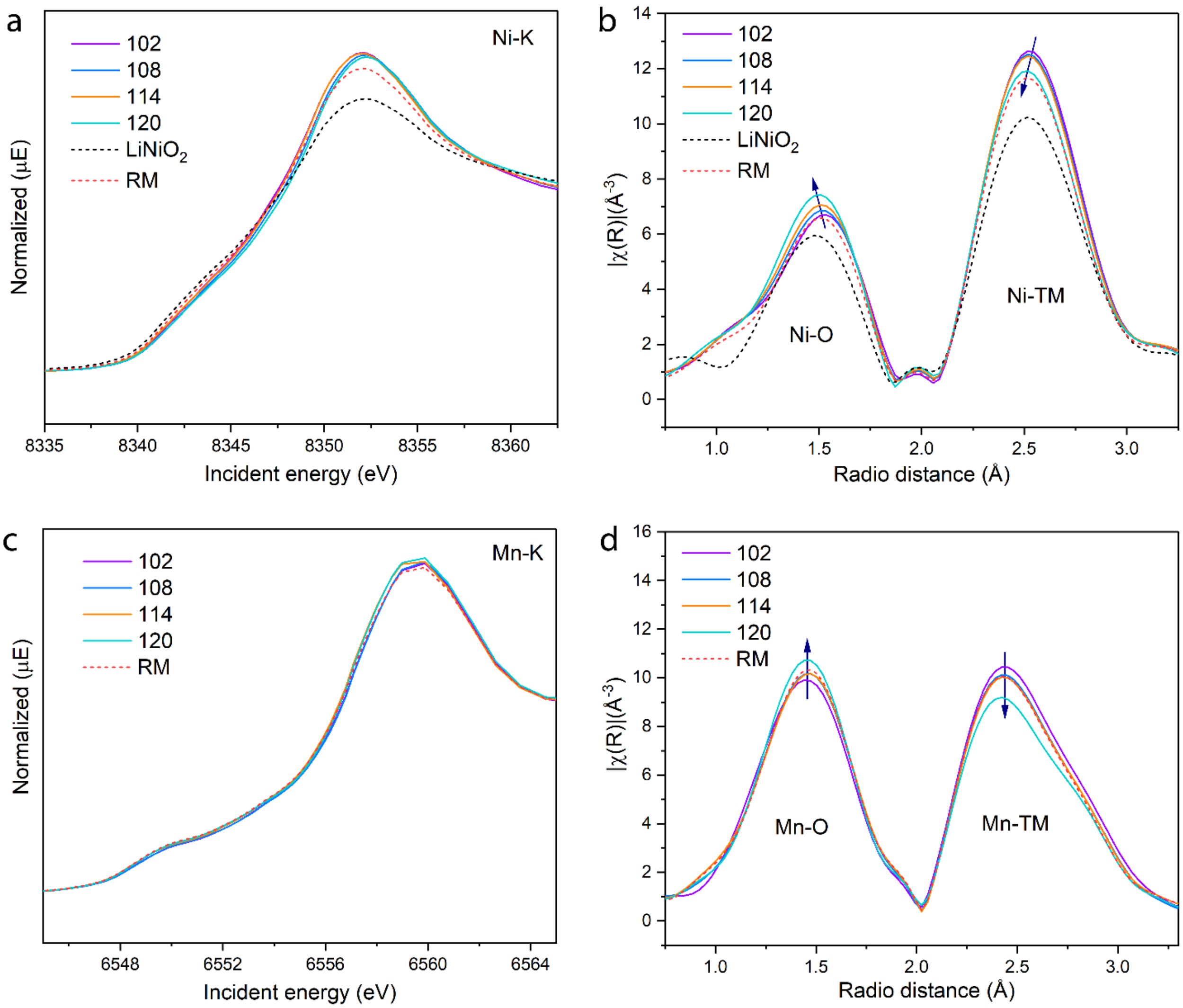

Fig. S2 The normalized K-edge X-ray absorption near edge structure (XANES) spectra of **a.** Ni and **c.** Mn and the corresponding Fourier transformed magnitude of k^3-weighted K-edge extended X-ray absorption fine structure (EXAFS) spectra of **b.** Ni and **d.** Mn.

Supplementary Note 2

In Fig. S2a, a slight shift to a higher energy of absorption edge is observed in HD-LNMO with larger Li content (especially between the edges of 102 and 120), indicating a higher valence state of Ni. The EXAFS spectra (Fig. S2b) exhibited a gradual shortening of Ni-O and Ni-TM bond lengths when Li content increased, which also corresponds to the gradual increase of the average Ni oxidation states. The above observations are in line with the decreased Ni/Li exchange from the combined refinement. In Fig. S2c, Mn K-edge spectra of HD-LNMO exhibit similar positions of the absorption edges, suggesting the same valence state of Mn for these materials. In the EXAFS spectra (Fig. S2d), the Mn-O peak intensity is gradually increasing, and the intensity of the Mn-TM peak decreases when Li content is increased. For the spectra of HD-LNMO-120, the first peak intensity is already higher than the second one. Such behavior indicates an excess Li in the TM layer participating in Mn-M coordination,[1,2] in good agreement with the increased value of Li in the TM layers in the refinement results. Note that this would also explain the same tendency in the Ni-EXAFS spectra. The fewer nuclear electrons of Li tend to lower the intensity of the Mn/Ni-TM bonds.

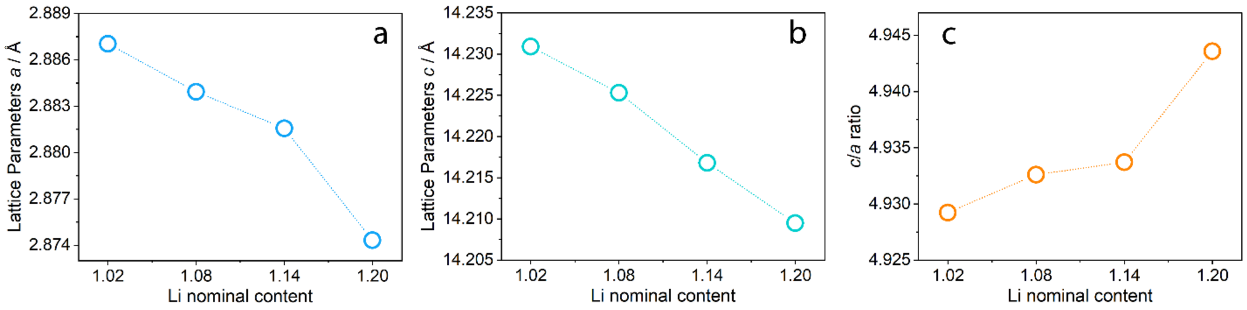

Fig.S3 Lattice parameters (from the combined refinement of XRD and ND data) as a function of Li content, including lattice parameters **a.** *a*, **b.** *c,* and **c.** *c*/*a*.

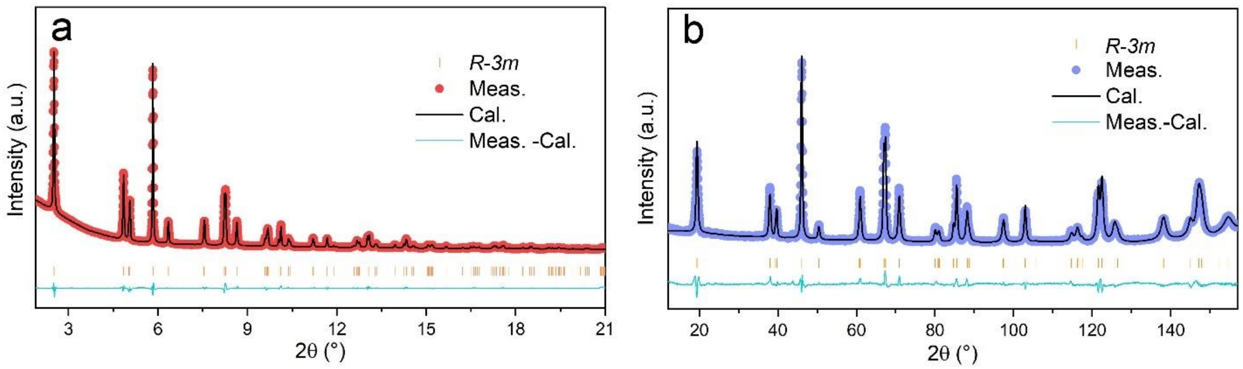

Fig. S4 a. Synchrotron XRD (λ = 0.2073 Å) and **b.** ND (λ = 1.594 Å) patterns and combined Rietveld refinement results of RM.

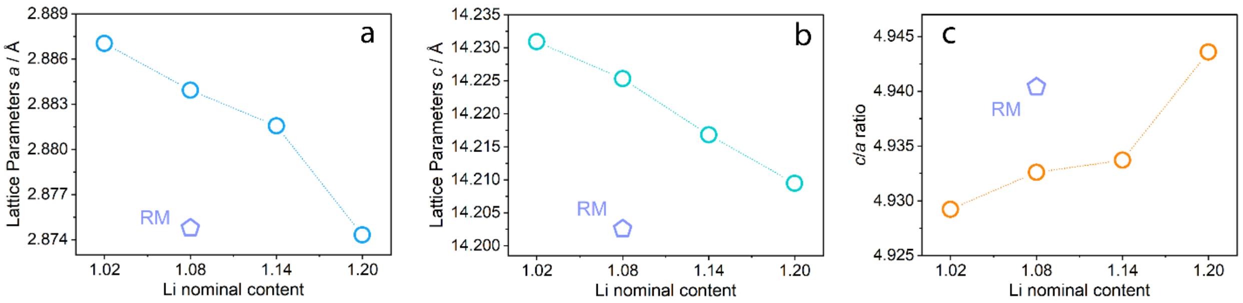

Fig. S5 The comparison of lattice parameters between HD-LNMO and RM. **a.** *a*, **b.** *c*, and **c.** *c/a*.

Supplementary Note 3

To check if the ^{6}Li enriched samples have the same structure and high purity as normal ^{7}Li samples, powder X-ray diffraction experiments on ^{6}Li enriched samples were performed (see below). All samples have a $NaFeO_2$-type layered structure. No other peaks are observed, confirming the high purity of the materials. The NMR spectra of normal ^{7}Li samples were also measured (see **Fig.S6**). The same features as ^{6}Li NMR spectra can be obtained, although the spinning sidebands appear. The intensity of peak B and peak D grow when Li content is increased. Such consistency proves that ^{6}Li enriched and non-enriched samples have the same structure and local environments.

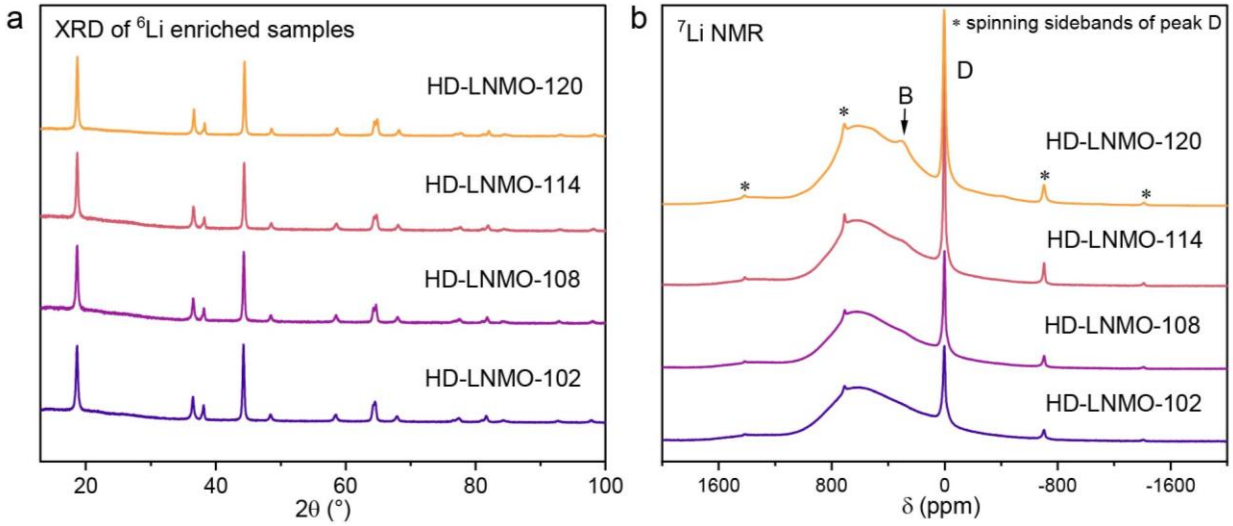

Fig.S6 a. XRD patterns of ^{6}Li enriched samples. **b.** ^{7}Li NMR spectra of normal samples (non-6Li enriched samples).

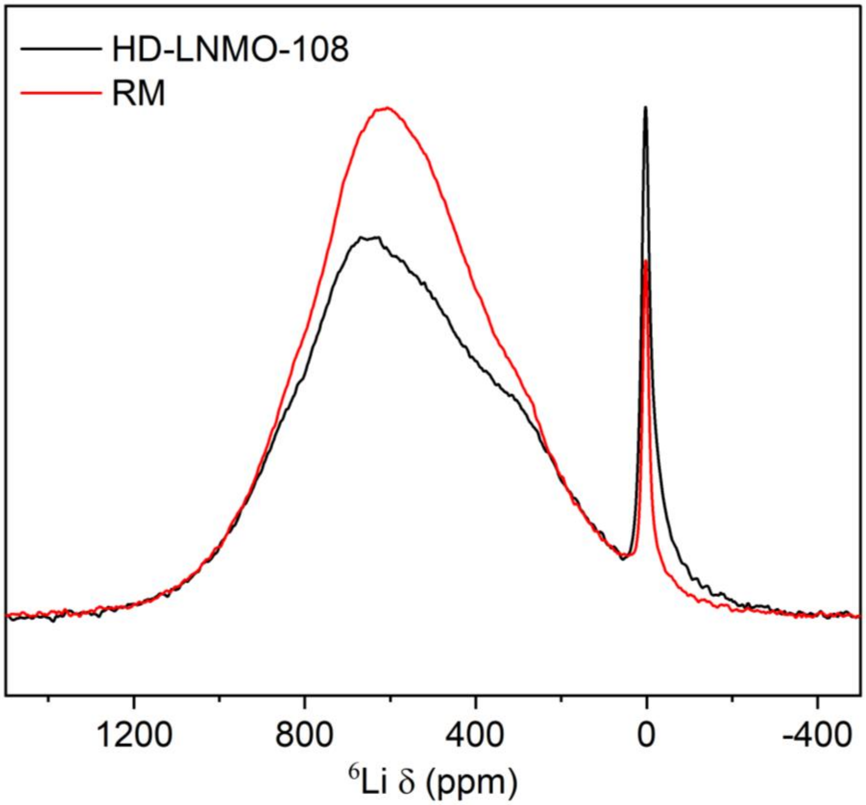

Fig. S7 The comparison of the ^{6}Li MAS-NMR spectra between HD-LNMO-108 and RM.

Supplementary Note 4

The STEM experiment was conducted on the 120 sample, and the corresponding HAADF image is shown in Fig. S8. Similar to the image of 108, an obvious decrease in the contrast of the TM layer is observed on the left side, indicating the replacement of Ni and Mn by Li. Two regions are marked (A and B) and both can be indexed to a layered structure (*R-3m*), as verified by the FFT patterns from the selected areas. However, the two domains exhibit different characteristic periodic atomic arrangements in the Li layers, as shown by the height profiles of lines 1 and 2. The contrast in the Li layers has been greatly increased from region A to region B, suggesting a higher Li/Ni exchange in region B.

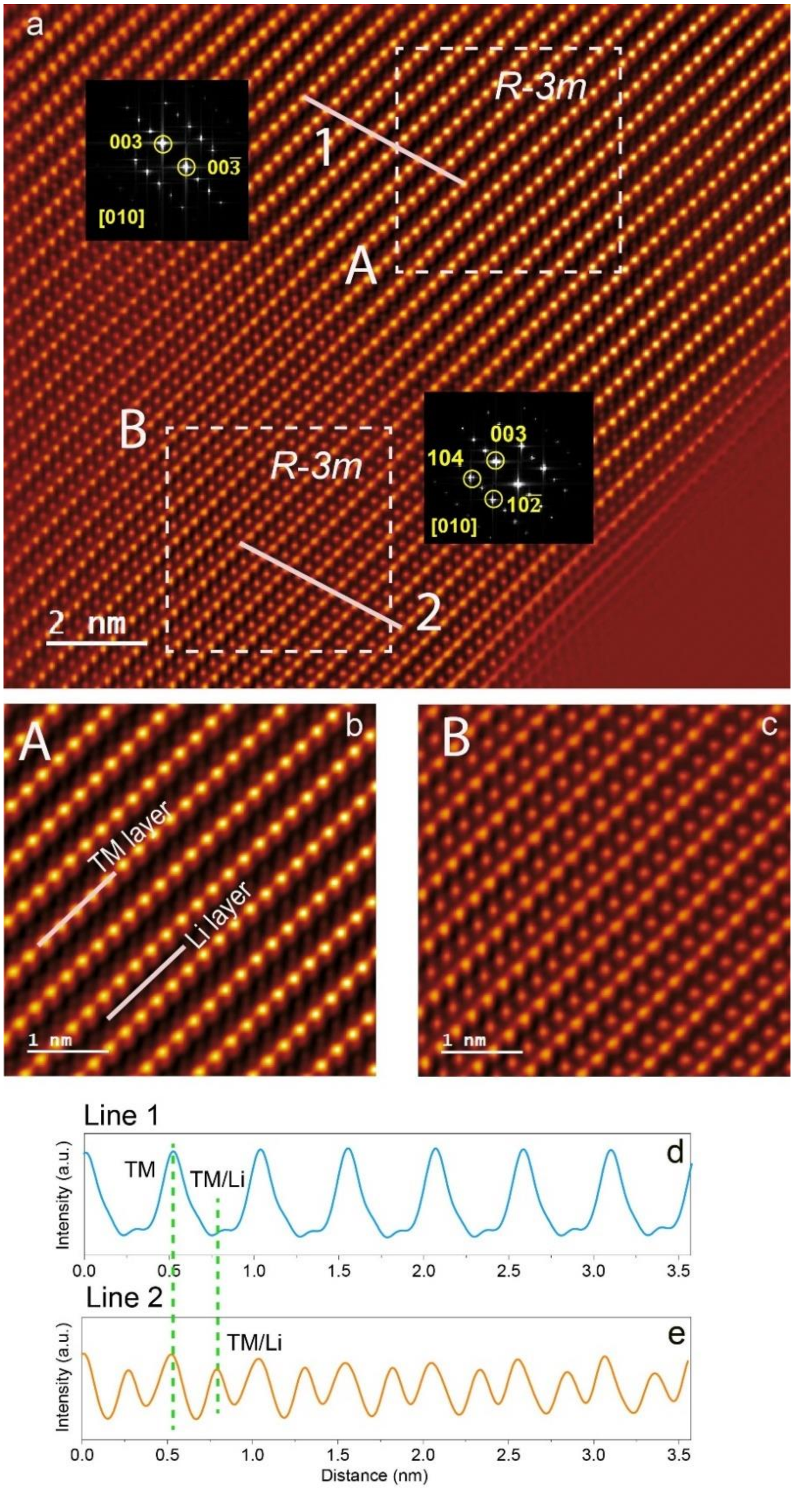

Fig. S8 a. HAADF STEM image showing the atomic structures of the HD-LNMO-120. The inset is the FFT patterns from the selected regions of A and B, corresponding to layered (*R-3m*) structures. **d.** and **e.** Line intensity profiles from lines 1 and line 2 in **a.** TM or Li are labeled based on the difference in the relative intensity. **b.** and **c.** Enlarged HAADF-STEM images from the marked regions A and B in **a.** TM and Li layers are indicated in **b.**

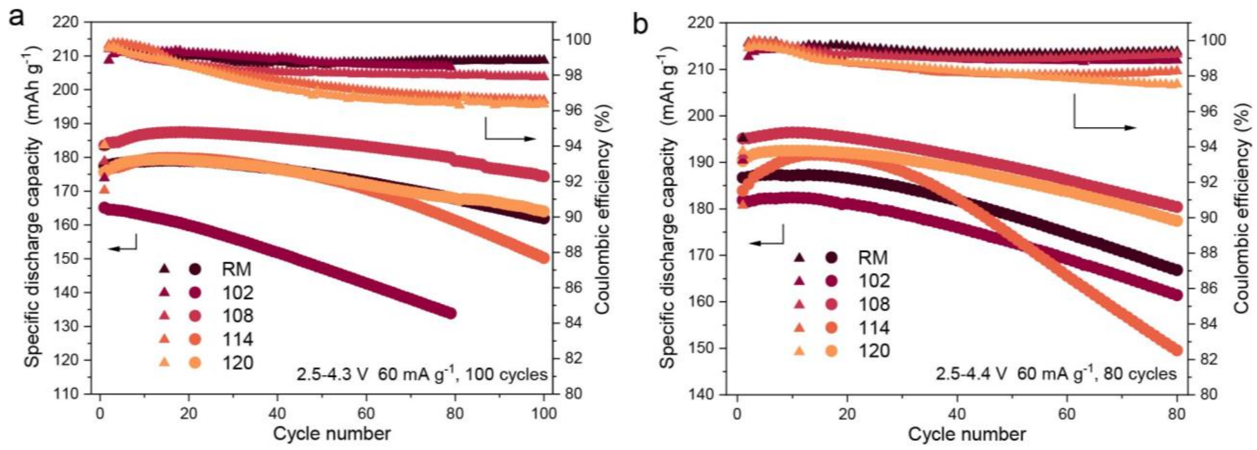

Fig. S9 Corresponding specific discharge capacity and Coulombic efficiency of HD-LNMO positive electrodes in half cells (at room temperature (25 °C), 18 mA g^{-1}, and with an active material mass loading of around 4 mg cm^{-2}) in the voltage ranges of **a.** 2.5-4.3 V and **b.** 2.5-4.4 V.

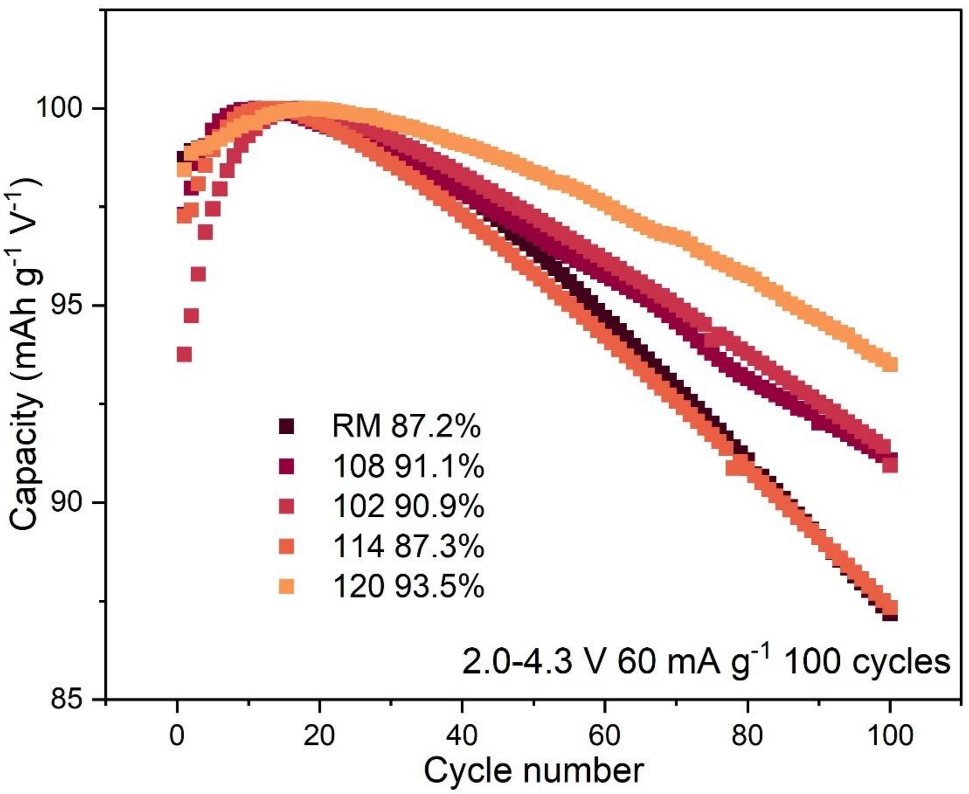

Fig. S10 Capacity retentions of HD-LNMO cathodes cycled at 25°C and 60 mA g^{-1} in the voltage range of 2.0-4.3 V

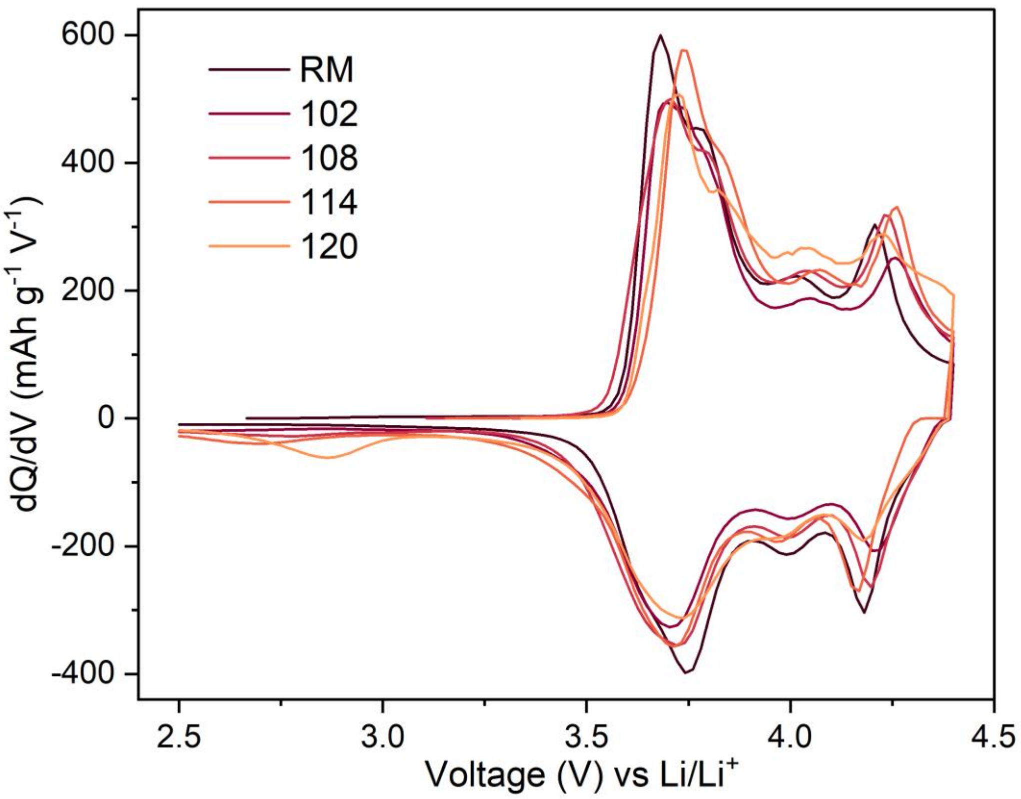

Fig. S11 The comparison of dQ/dV profiles between HD-LNMO and RM cathodes (at 25°C and 60 mA g^{-1}, 1st cycle, 2.5-4.4 V).

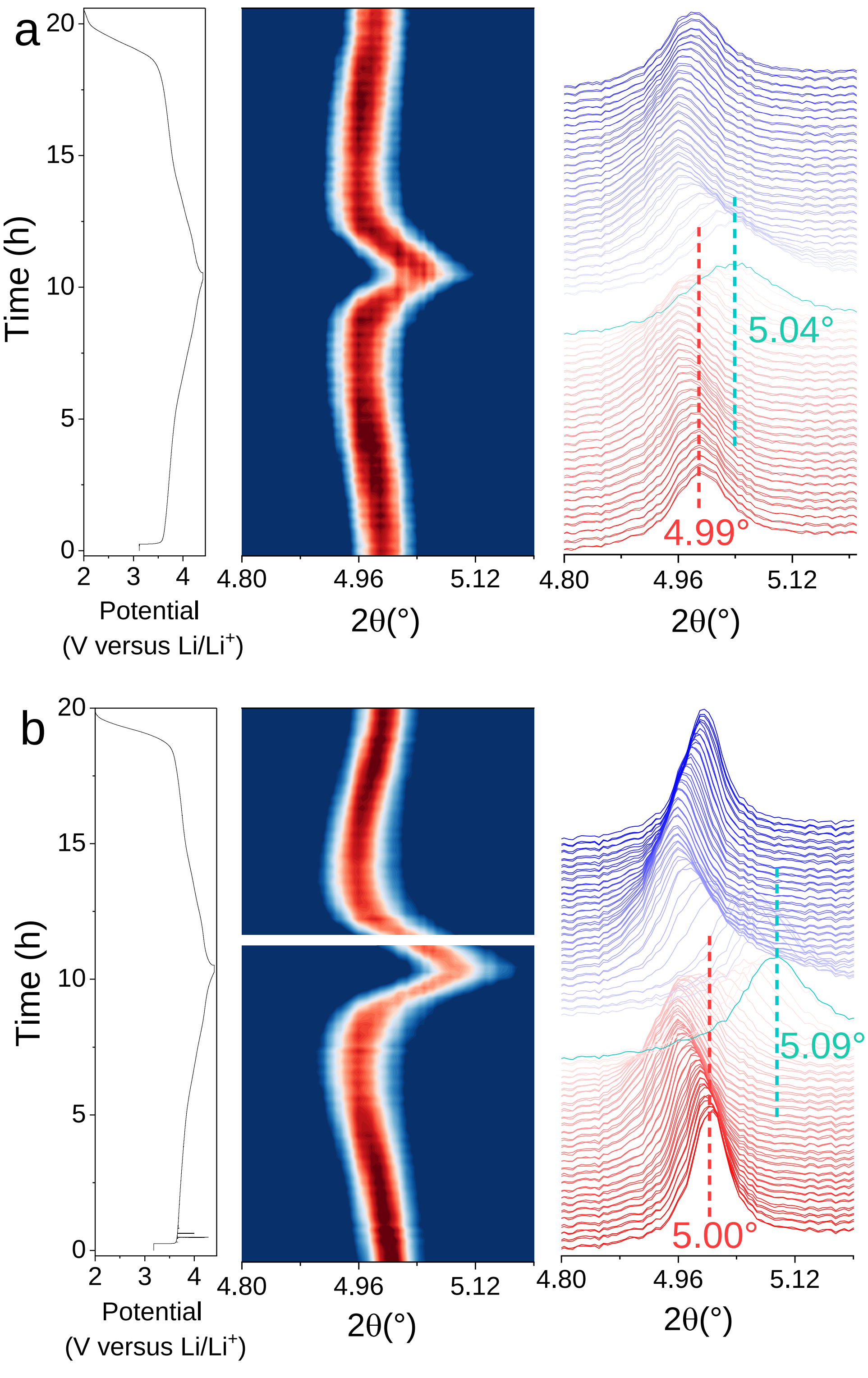

Fig. S12 2D contour pattern of the *in situ* SXRD of 102 (**a.**) and RM (**b.**) cathodes. The corresponding SXRD patterns are displayed on the left side (λ = 0.4139 Å). The location of the 003 reflections in the pristine state and SoC 81% are labeled.

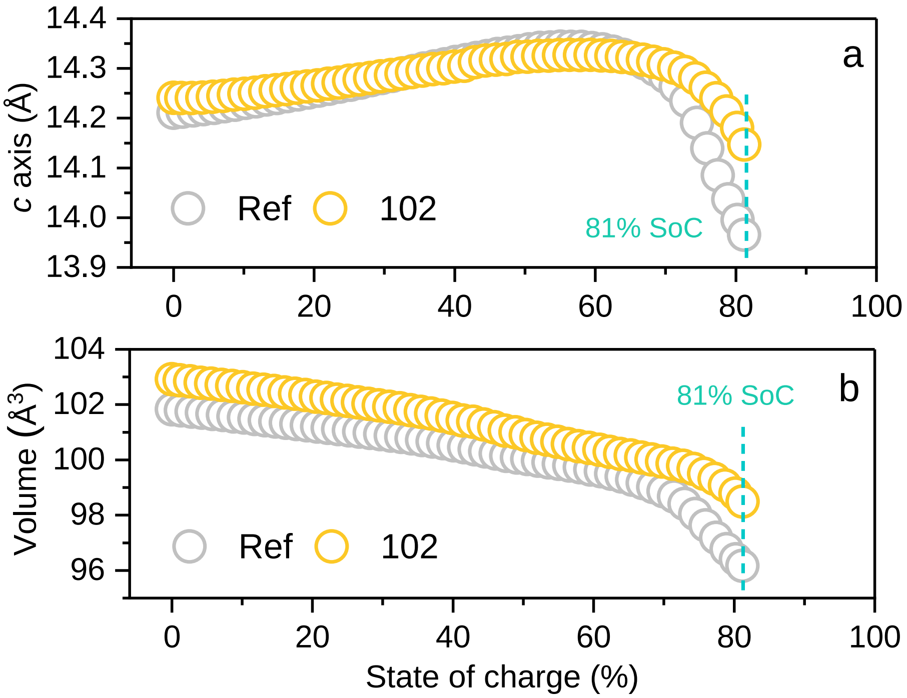

Fig. S13 *c*-lattice parameters and lattice volume changes of **a.** HD-LNMO-102 and **b.** RM during charge.

Supplementary Note 5

Since there is an obvious difference in electrochemical performance between 108, 114, and 120, a detailed structural analysis was conducted based on the *in situ* XRD patterns (Fig. S14). For each material, three patterns were chosen (pristine, charged states (81% SoC), and fully discharged state). The bond lengths and Bond Valence Calculation (BVS) information are displayed in the table below based on the Rietveld refinement results.

For the bond length, the Ni-O bond decreases owing to the oxidation of Ni^{3+}, and the Li-O bond is enlarged correspondingly. After discharge, all bond lengths can return to values similar to the pristine states. Regarding the BVS calculation, the predicted oxidation states of Ni at TM layers are 2.95, 2.86, and 2.87 for 108, 114, and 120, respectively. Based on the edge position of Ni in XANES, the order for the oxidation states of Ni is 120>108>114. The difference originates from the fact that the XANES results represent all Ni in the materials and the BVS predicts the oxidation states of Ni in TM layers. This can be further proved by the consistency between the two methods in the oxidation states of Mn: the oxidation states of Mn are predicted to be 3.54, 3.42, and 3.44 for 108, 114, and 120, respectively. They are in line with the Mn K-edge XANES results because Mn is believed to be only situated in the TM layers. Therefore, the BVS values for Ni in the TM layers provide further structural information for pristine states. Most of the Ni ions in TM layers of 108 are Ni^{3+} and the numbers of Ni^{3+} ions are decreased for 114 and 120.

	A	B	C	D	E	F	G	H	I	J	K
1		Ni-O bond	Li-O bond	Li@Li	Ni@Li	Li@TM	Ni@TM	Mn@TM	Nb@TM	Mo@TM	W@TM
2	108-pristine	1.9482(15)	2.1525(19)	0.938(2)	1.560(3)	1.630(3)	2.954(5)	3.540(6)	5.426(9)	5.368(9)	5.515(9)
3	108-charge (81% SoC)	1.8758(21)	2.1833(28)	0.863(3)	1.435(4)	1.982(5)	3.592(8)	4.305(10)	6.598(15)	6.527(15)	6.706(15)
4	108-discharge	1.9341(15)	2.1724(18)	0.889(2)	1.478(3)	1.693(3)	3.069(5)	3.678(6)	5.637(9)	5.577(9)	5.730(9)
5											
6	114-pristine	1.9608(15)	2.1339(18)	0.987(2)	1.640(3)	1.575(3)	2.855(5)	3.422(6)	5.244(9)	5.188(9)	5.330(9)
7	114-charge (81% SoC)	1.8874(21)	2.1637(28)	0.910(3)	1.513(5)	1.921(5)	3.481(8)	4.172(10)	6.395(10)	6.326(15)	6.499(15)
8	114-discharge	1.9504(15)	2.1474(18)	0.951(2)	1.581(3)	1.620(3)	2.936(5)	3.519(6)	5.394(9)	5.336(9)	5.482(9)
9											
10	120-pristine	1.9592(23)	2.1257(27)	1.009(3)	1.677(5)	1.582(4)	2.868(7)	3.437(9)	5.268(13)	5.211(13)	5.354(13)
11	120-charge (81% SoC)	1.8897(29)	2.1696(38)	0.896(4)	1.489(6)	1.909(6)	3.460(11)	4.147(13)	6.536(20)	6.288(20)	6.460(21)
12	120-discharge	1.9400(22)	2.1465(27)	0.954(3)	1.585(5)	1.666(4)	3.020(7)	3.620(9)	5.548(14)	5.488(14)	5.638(14)

Fig. S14 The bond length and BVS information of HD-LNMO-108, 114, and 120. For each material, three patterns were chosen (pristine, charged states (81% SoC), and fully discharged state).

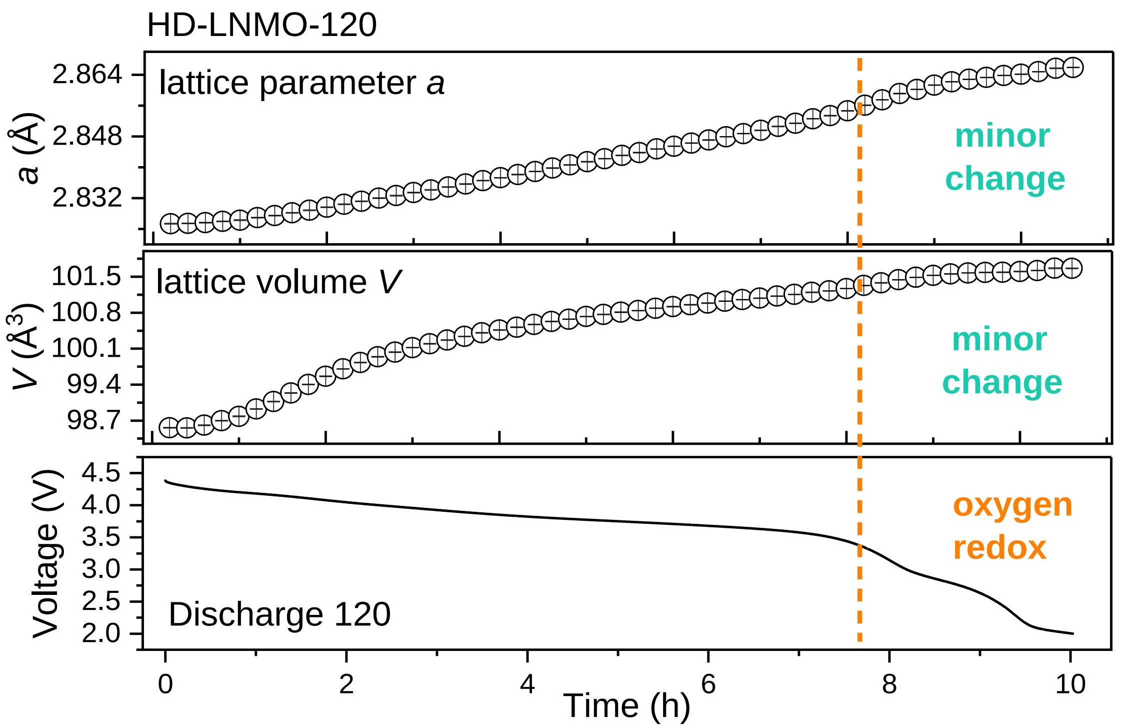

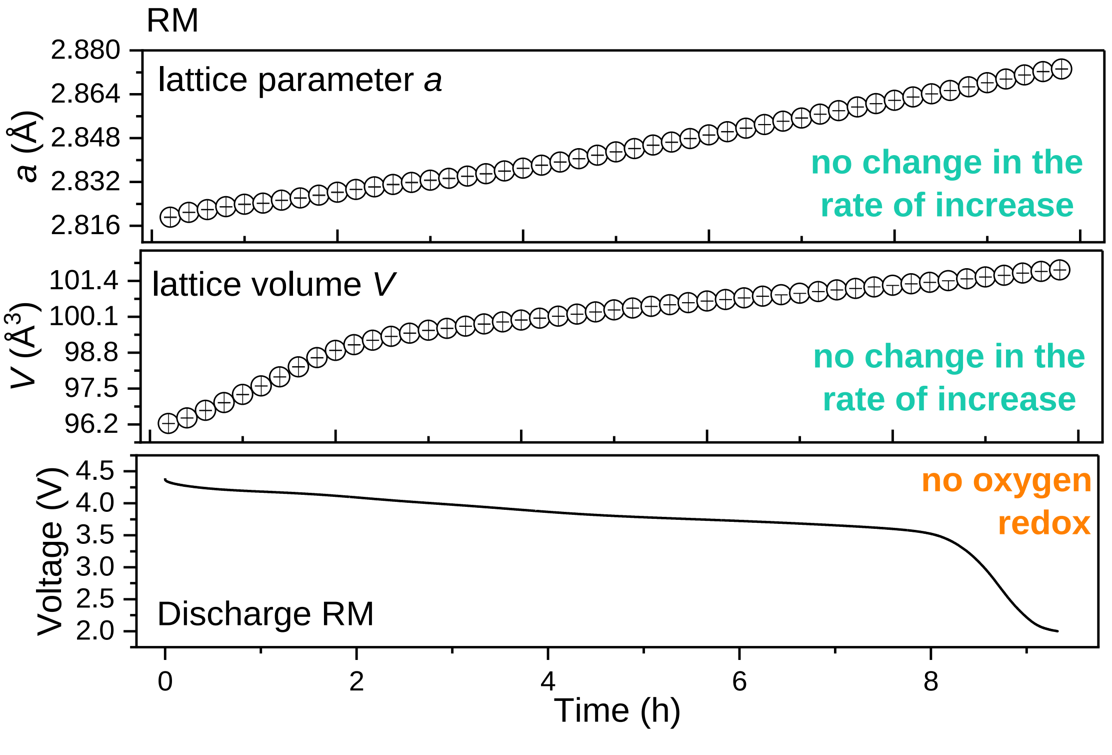

Fig. S15 changes in the *a*-axis lattice parameter (Δc) and the lattice volume (ΔV) of RM and 120 during 1st discharging period based on the Rietveld refinement results of the in situ XRD patterns.

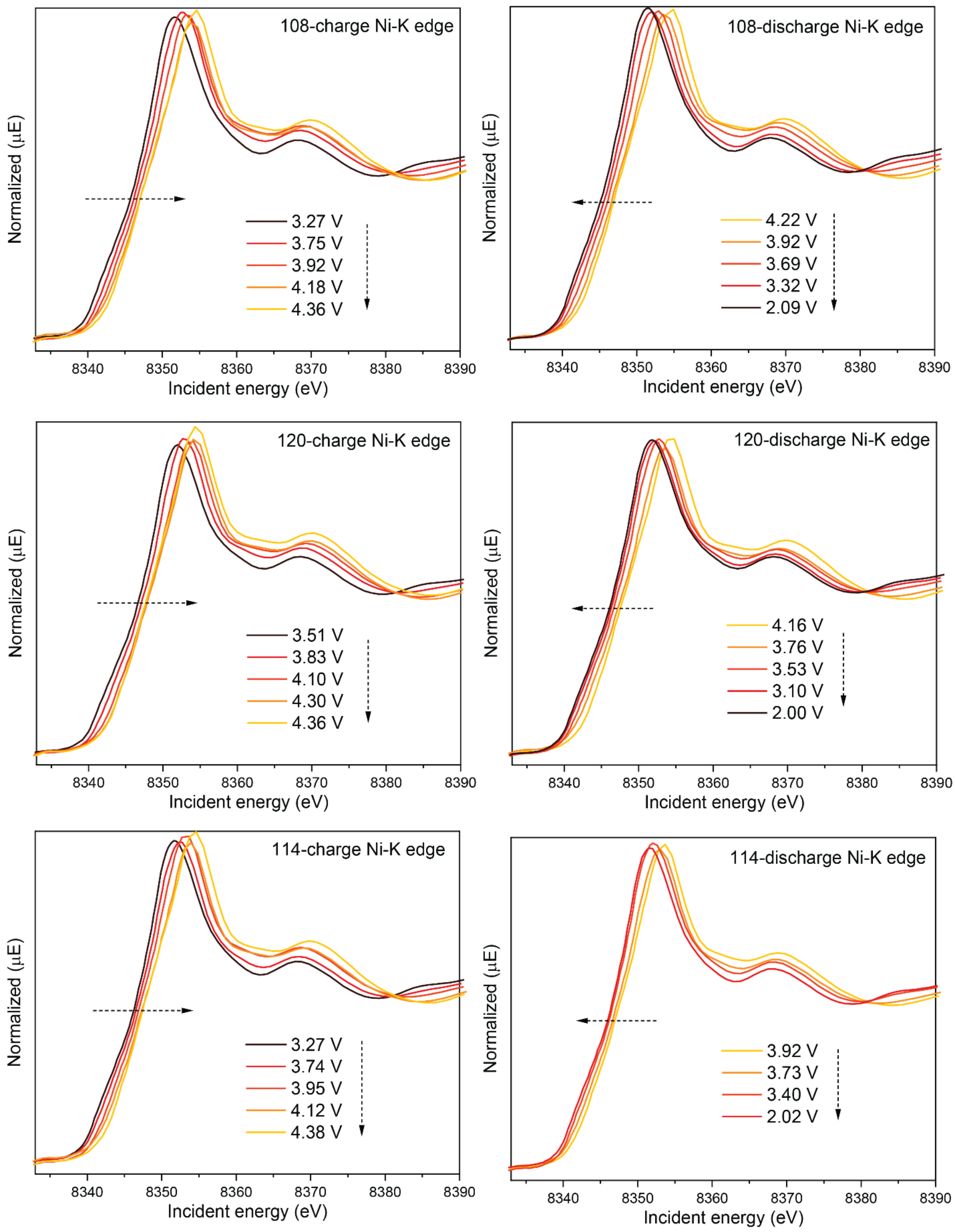

Fig. S16 selected in situ XANES data at the Ni K-edge of HD-LNMO-108,114, and 120 during the 1st cycle.

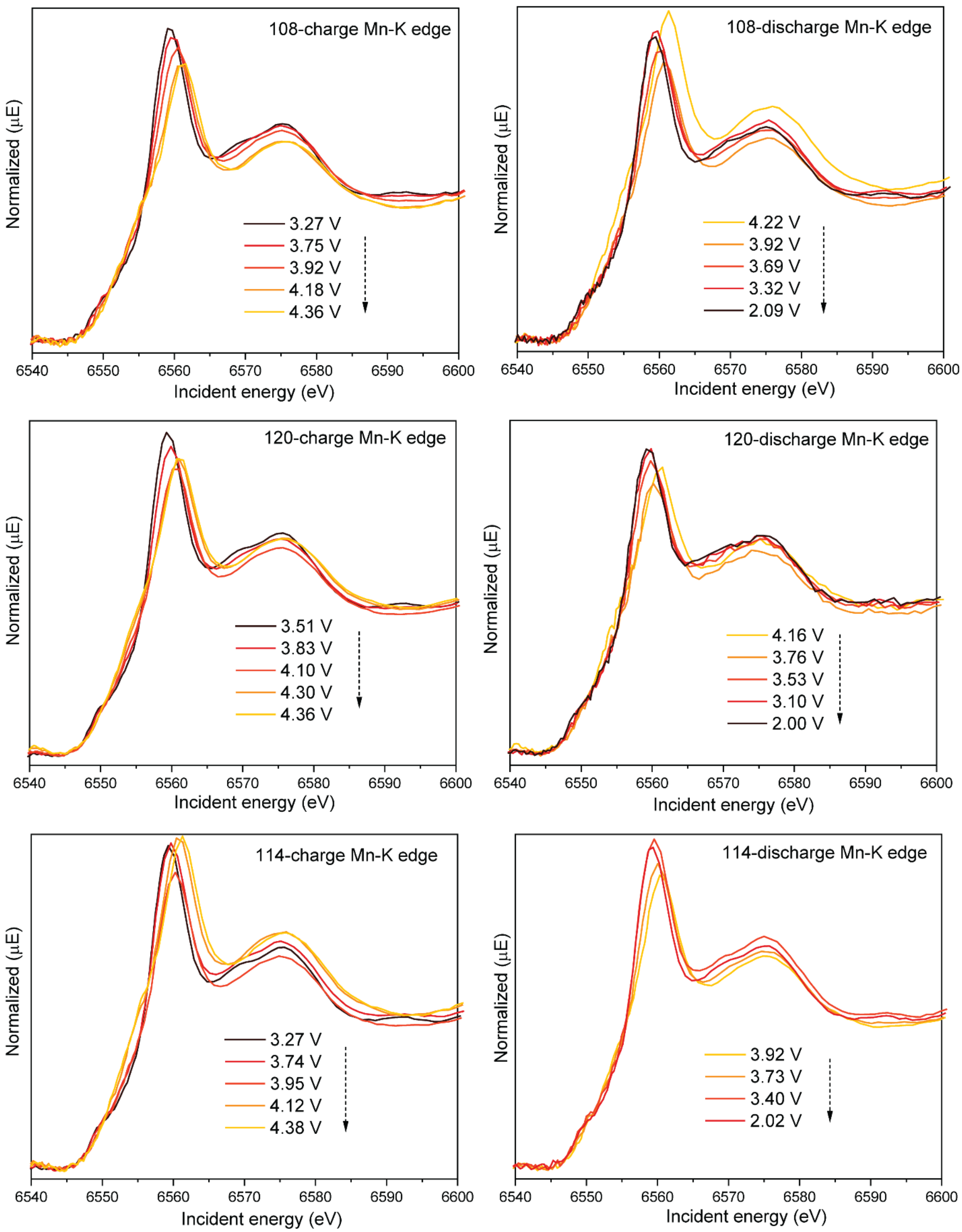

Fig. S17 selected in situ XANES data at the Mn K-edge of HD-LNMO-108,114, and 120 during the 1st cycle.

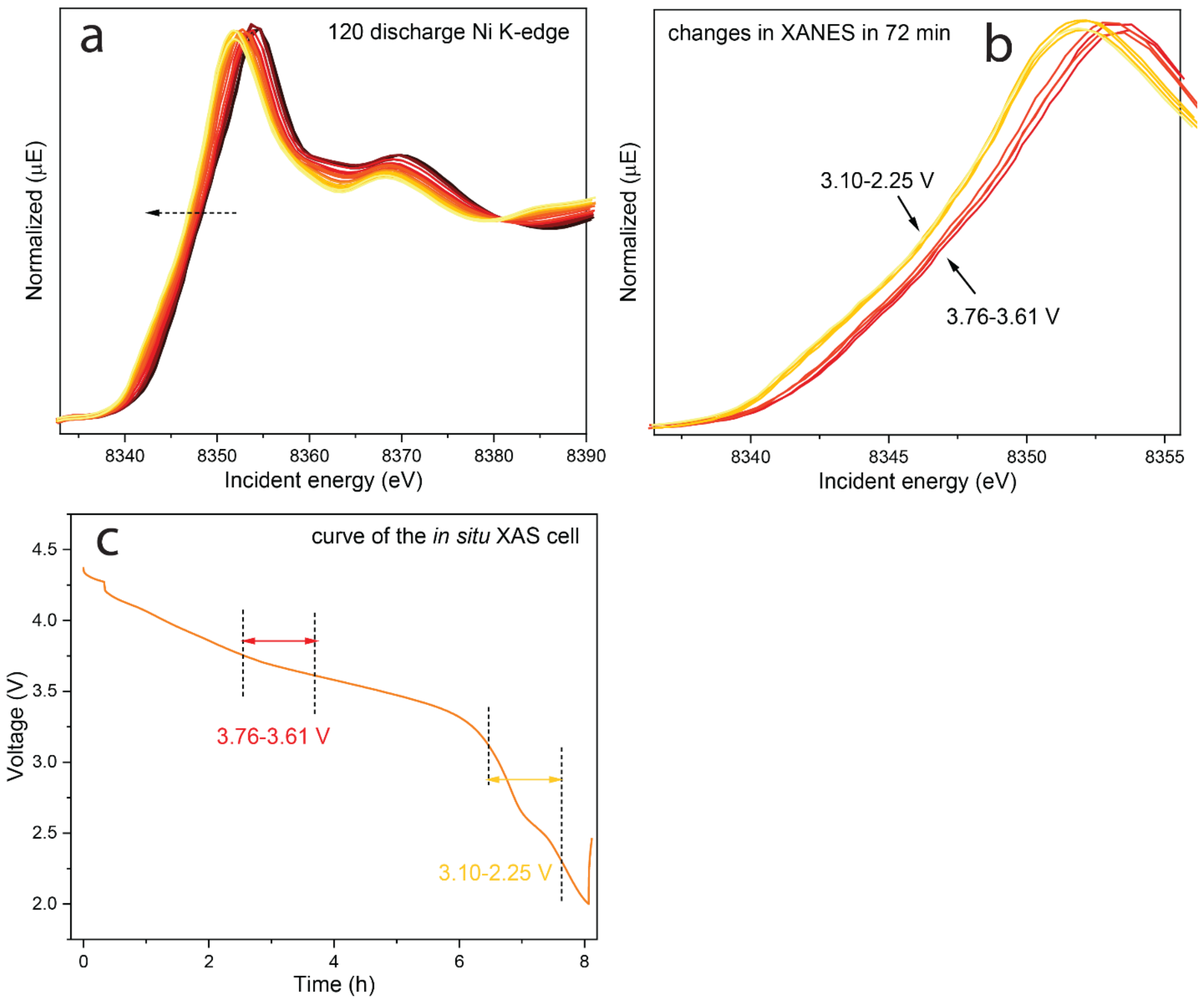

Fig. S18 a. in situ XANES data at the Ni K-edge of HD-LNMO-120 during the discharge at the 1st cycle, **b.** the comparison of the XANES data at the two selected periods, and **c.** the corresponding electrochemical curve of the in situ cell.

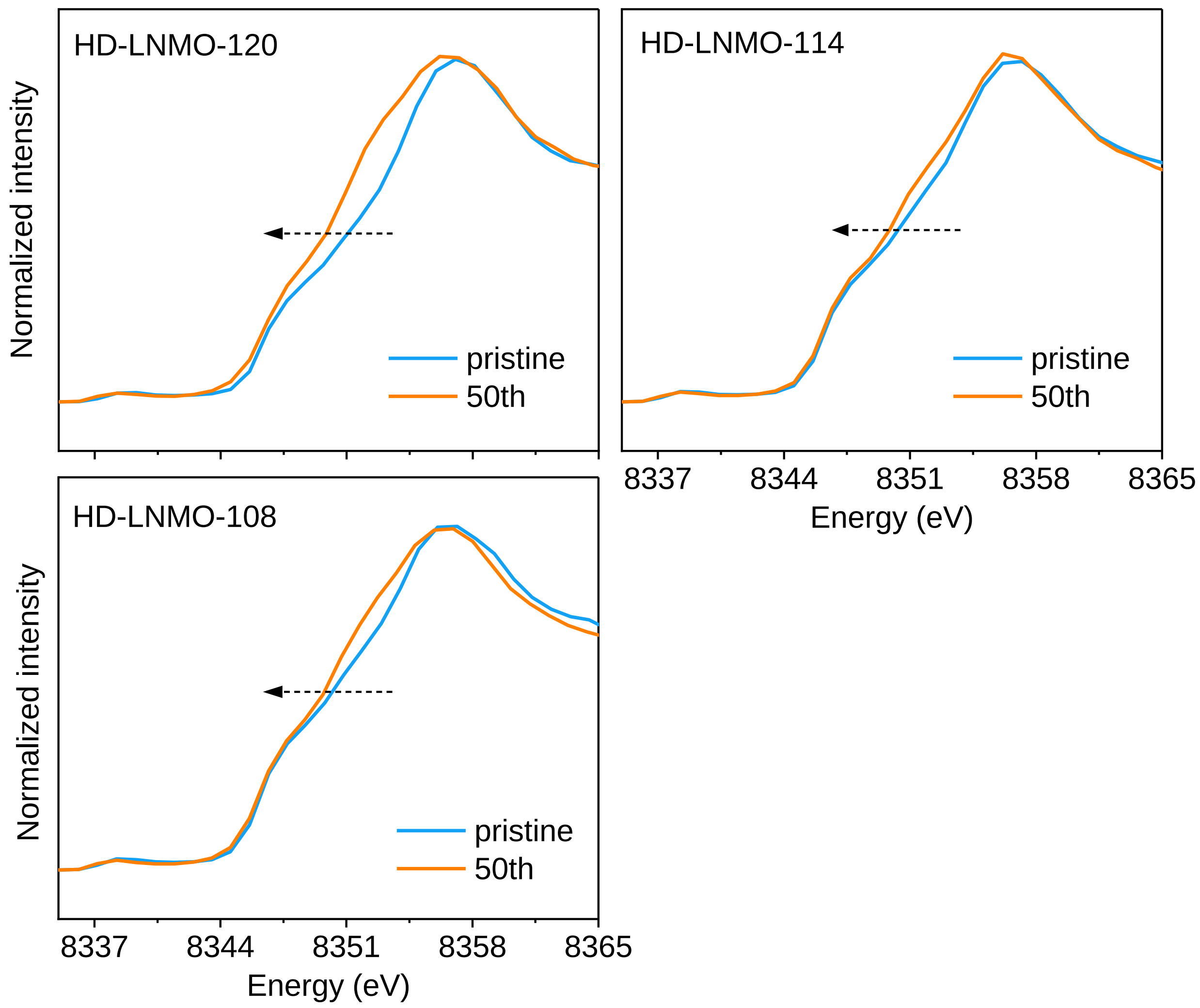

Fig. S19 Ex situ hard XAS spectra at the Ni K-edge on pristine and cycled electrodes after 50 cycles (60 mA g^{-1} and 25°C, 2.5-4.4 V) for HD-LNMO-108, 114, and 120.

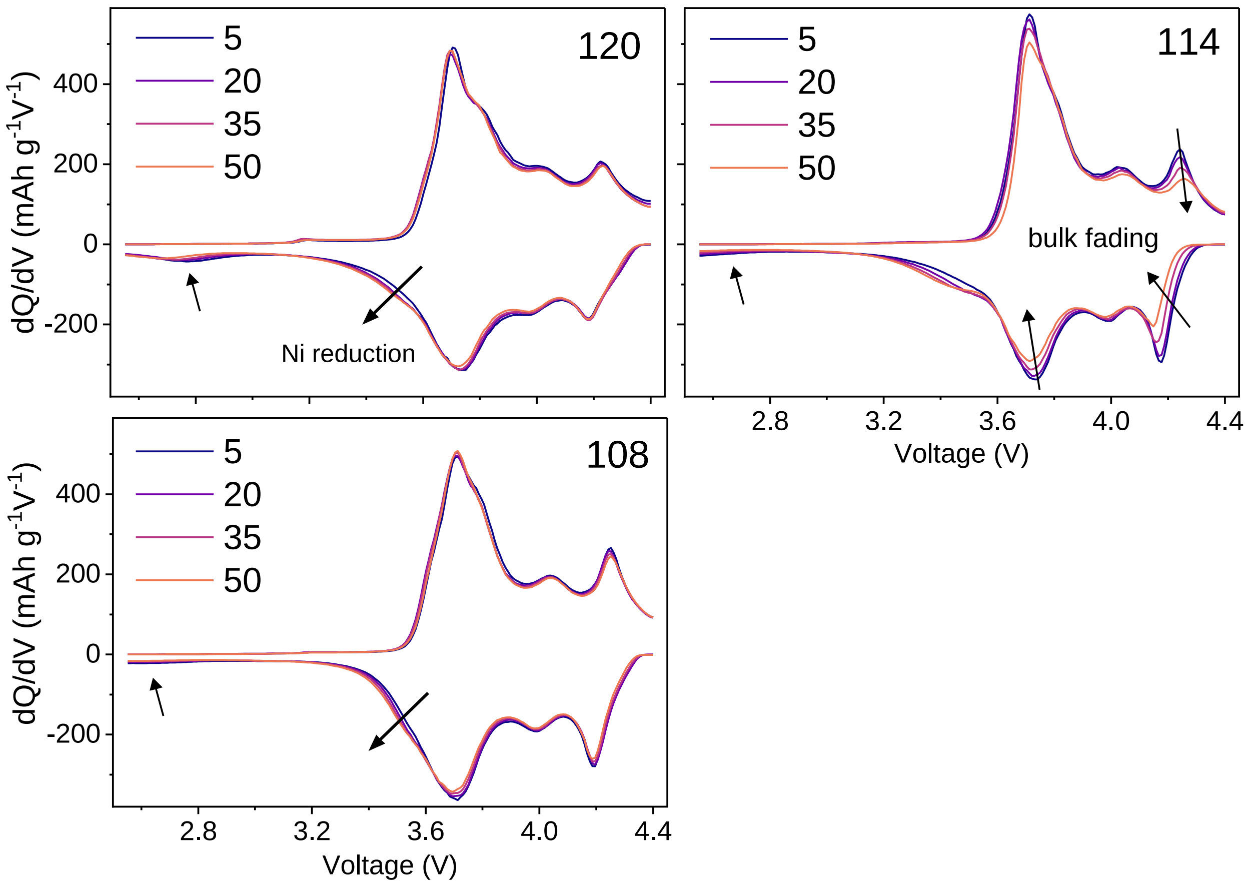

Fig. S20 The dQ/dV profiles after every 15 cycles of 108, 114, and 120 when cycled in half cells in the voltage range of 2.5-4.4 V.

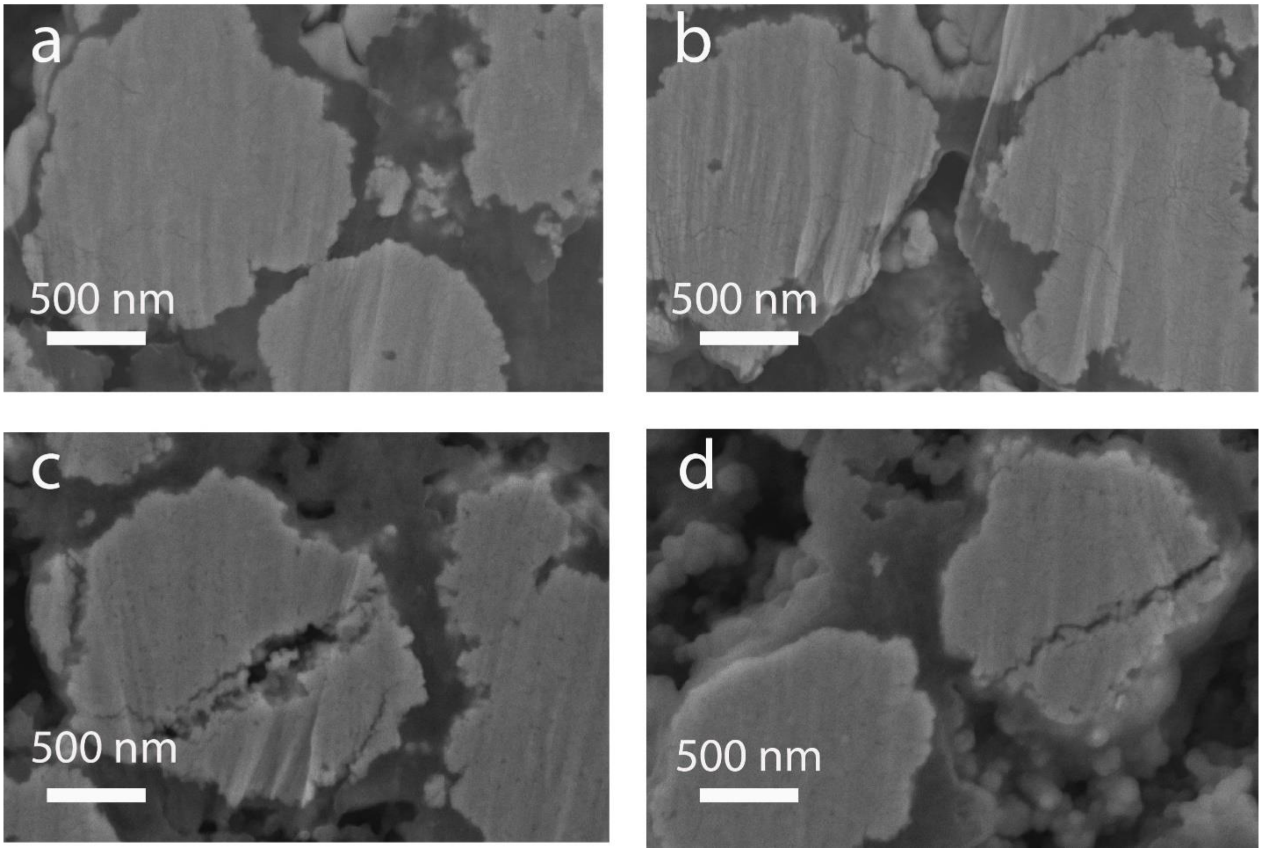

Fig. S21 the cross-section SEM images of cycled 108 (**a.** and **b.**) and 114 (**c.** and **d.**) electrodes.

Table S1 ICP-OES determined compositions of HD-LNMO and reference materials.

Nominal Li content	Li	Ni	Mn
1.02	1.025±0.010	0.825±0.015	0.118±0.002
1.08	1.052±0.009	0.802±0.015	0.114±0.003
1.14	1.090±0.010	0.769±0.015	0.110±0.002
1.20	1.134±0.012	0.730±0.020	0.106±0.003
Reference	1.060±0.010	0.777±0.015	0.124±0.003

Nominal Li content	Mo	Nb	W	Co
1.02	0.0171±0.0005	0.00632±0.00027	0.00935±0.00022	0
1.08	0.0166±0.0003	0.00620±0.00017	0.00916±0.00032	0
1.14	0.0160±0.0002	0.00596±0.00016	0.00875±0.00010	0
1.20	0.0155±0.0004	0.00580±0.00012	0.00852±0.00021	0
Reference	0	0	0	0.0376±0.0005

Table S2 Crystallographic data of HD-LNMO-102 determined from the combined refinement of SXRD and ND patterns.

HD-LNMO-102	χ^2 = 3.10, Bragg R-factor 3.17% for ND and 2.17 for SXRD				
Space group $R\bar{3}m$	*a* = 2.87704(9) Å, *c* = 14.23092(84) Å *V* = 102.724(8)				
Atom	Site	x/a	y/b	z/c	Occupancy
O1	6*c*	0	0	0.25809(36)	1.000
Li1	3*b*	0	0	0.5	0.912(3)
Ni2	3*b*	0	0	0.5	0.088(3)
Ni1	3*a*	0	0	0	0.737(3)
Li2	3*a*	0	0	0	0.113(3)
Mn1	3*a*	0	0	0	0.118
Mo	3*a*	0	0	0	0.017
Nb1	3*a*	0	0	0	0.006
W1	3*a*	0	0	0	0.009

Table S3 Crystallographic data of HD-LNMO-108 determined from the combined refinement of SXRD and ND patterns.

HD-LNMO-108	χ^2 = 1.96, Bragg R-factor 2.76% for ND and 2.38 for SXRD				
Space group $R\bar{3}m$	*a* = 2.88393(8) Å, *c* = 14.22531(79) Å *V* = 102.462(8)				
Atom	Site	x/a	y/b	z/c	Occupancy
O1	6*c*	0	0	0.25813(34)	1.000
Li1	3*b*	0	0	0.5	0.933(3)
Ni2	3*b*	0	0	0.5	0.067(3)
Ni1	3*a*	0	0	0	0.735(3)
Li2	3*a*	0	0	0	0.119(3)
Mn1	3*a*	0	0	0	0.114
Mo	3*a*	0	0	0	0.017
Nb1	3*a*	0	0	0	0.006
W1	3*a*	0	0	0	0.009

Table S4 Crystallographic data of HD-LNMO-114 determined from the combined refinement of SXRD and ND patterns.

HD-LNMO-114	χ^2 = 4.61, Bragg R-factor 3.11% for ND and 2.40 for SXRD				
Space group $R\bar{3}m$	*a* = 2.88156(10) Å, *c* = 14.21680(90) Å *V* = 102.233(8)				
Atom	Site	x/a	y/b	z/c	Occupancy
O1	6*c*	0	0	0.25842(38)	1.000
Li1	3*b*	0	0	0.5	0.949(4)
Ni2	3*b*	0	0	0.5	0.051(4)
Ni1	3*a*	0	0	0	0.719(4)
Li2	3*a*	0	0	0	0.141(4)
Mn1	3*a*	0	0	0	0.110
Mo	3*a*	0	0	0	0.016
Nb1	3*a*	0	0	0	0.006
W1	3*a*	0	0	0	0.009

Table S5 Crystallographic data of HD-LNMO-120 determined from the combined refinement of SXRD and ND patterns.

HD-LNMO-120	χ^2 = 4.95, Bragg R-factor 3.05% for ND and 2.75 for SXRD				
Space group $R\bar{3}m$	*a* = 2.87432(9) Å, *c* = 14.20948(76) Å *V* = 101.667(8)				
Atom	Site	x/a	y/b	z/c	Occupancy
O1	6*c*	0	0	0.25843(33)	1.000
Li1	3*b*	0	0	0.5	0.965(4)
Ni2	3*b*	0	0	0.5	0.035(4)
Ni1	3*a*	0	0	0	0.696(4)
Li2	3*a*	0	0	0	0.169(4)
Mn1	3*a*	0	0	0	0.106
Mo	3*a*	0	0	0	0.016
Nb1	3*a*	0	0	0	0.006
W1	3*a*	0	0	0	0.008

Table S6 Crystallographic data of RM determined from the combined refinement of SXRD and ND patterns.

RM χ^2 = 4.60, Bragg R-factor 3.41% for ND and 2.42 for SXRD					
Space group $R\bar{3}m$ a = 2.87477(8) Å, c = 14.20255(71) Å V = 101.649(8)					
Atom	Site	x/a	y/b	z/c	Occupancy
O1	6*c*	0	0	0.25838(37)	1.000
Li1	3*b*	0	0	0.5	0.959(4)
Ni2	3*b*	0	0	0.5	0.041(4)
Ni1	3*a*	0	0	0	0.736(4)
Li2	3*a*	0	0	0	0.101(4)
Mn1	3*a*	0	0	0	0.124
Co	3*a*	0	0	0	0.038

Table S7 Detailed fitting parameters and results for ^{6}Li NMR

	Peak	Shift (ppm)	Width (ppm)	G/L ratio	Ratio (%)
HD-LNMO-102	A	678	469	0.70	73.7
	B	305	72	0.20	0.3
	C	310	425	1	18.6
	D	3	22	0.20	4.8
	E	-31	71	0.20	2.6

	Peak	Shift (ppm)	Width (ppm)	G/L ratio	Ratio (%)
HD-LNMO-108	A	653	441	0.80	68.7
	B	296	113	0.20	1.4
	C	302	418	1	21.1
	D	2	21	0.20	5.0
	E	-33	77	0.20	3.7

	Peak	Shift (ppm)	Width (ppm)	G/L ratio	Ratio (%)
HD-LNMO-114	A	636	443	0.90	63.3
	B	291	120	0.40	2.0
	C	294	380	0.40	26.4
	D	3	21	0.20	5.6
	E	-26	60	0.40	2.8

	Peak	Shift (ppm)	Width (ppm)	G/L ratio	Ratio (%)
HD-LNMO-120	A	605	441	1	63.4
	B	296	111	0.20	4.3
	C	260	387	1	22.4
	D	4	20	0.20	7.1
	E	-21	69	0.20	2.6

Supplementary References

1. Yin, C. *et al.* Structural insights into composition design of Li-rich layered cathode materials for high-energy rechargeable battery. *Mater. Today* **51**, 15–26 (2021).

2. Liu, W. *et al.* Enhancement of La–Ti modification on the conductivity and stability of Co-free Li- and Mn-rich layered cathode. *J. Power Sources* **613**, 234805 (2024).
